# Supplementary material for: Real-time quasi-distributed fiber optic sensor based on resonance frequency mapping
Source: Sci Rep. 2019 Mar 8;9:3921. doi: 10.1038/s41598-019-40472-2 (PMC6408579; doi:10.1038/s41598-019-40472-2)
Supplement: Supplementary file 2 — Supplementary info [file 41598_2019_40472_MOESM2_ESM.docx]

**Real-time quasi-distributed fiber optic sensor based on resonance frequency mapping**

**Gyeong Hun Kim^1^, Sang Min Park^1^, Chang Hyun Park^1^, Hansol Jang^1^,**

**Chang-Seok Kim^1*^, Hwi Don Lee^2*^**

^1^Department of Cogno-Mechatronics Engineering, Pusan National University, Busan 46241, Korea

^2^Advanced Photonics Research Institute, Gwangju Institute of Science and Technology, Gwangju 61005, Korea

* Correspondence and requests for materials should be addressed to C.-S. K. (ckim@pusan.ac.kr) or H.D. L.

([rahido@gist.ac.kr](mailto:rahido@gist.ac.kr)).

**Supplementary Figures**


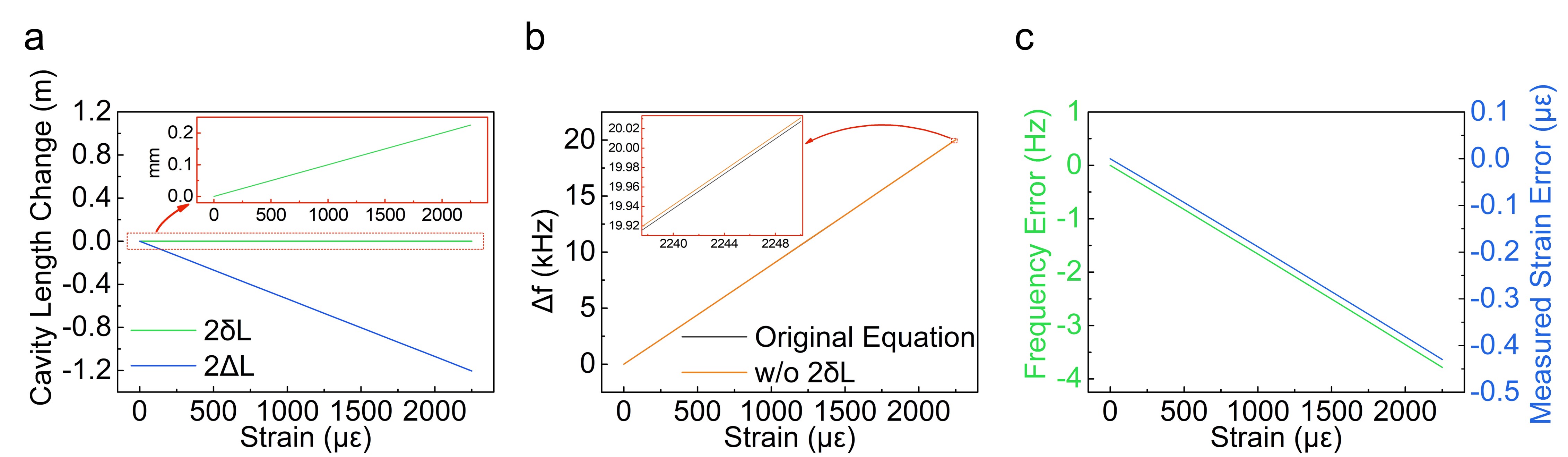


**Supplementary Figure S1 |** (a) Simulation of the change in total cavity length induced by the physical strain of the sensing FBG (*2δL*) and the shift in the reflection point on the CFBG *(2ΔL*). The strain-holder length was 100 mm and the dispersion of the CFBG was −2152 ps·nm^−1^. In the simulation result, the ratio of the two types of change in the total cavity length (*ΔL/δL)* was 5351. (b) Comparison of changes in resonance frequency using Eq. 4 and the equation without the *2δL* variable. (c) Error in resonance frequency owing to the exclusion of *δL* and the measured strain error according to the approximated equation (Eq. 5). The overall strain error was less than 1 με, which was less than the measurement stability of this system (2.4 με). As a consequence of these results, *δL* was sufficiently small to be regarded as negligible.

**
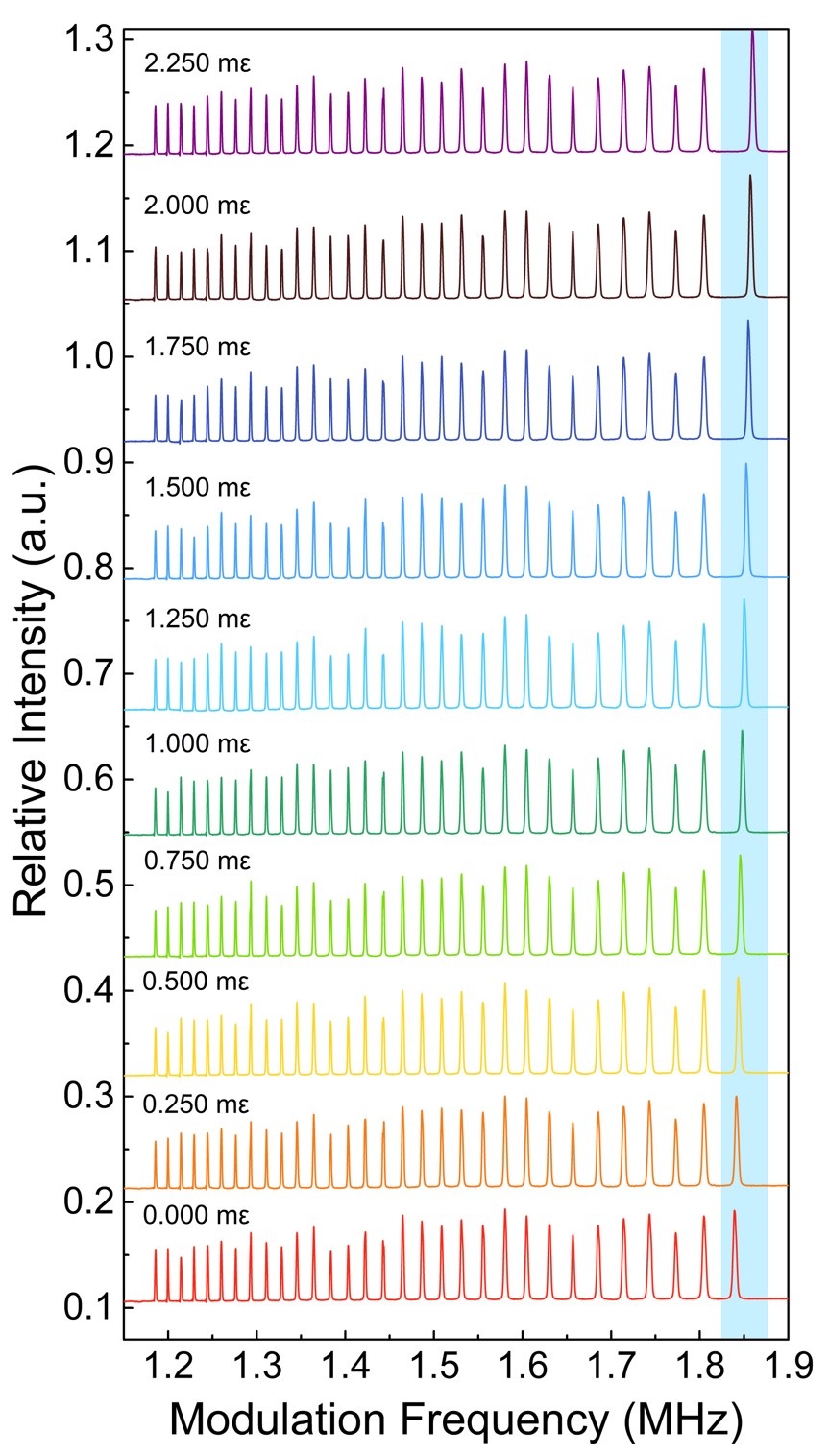
**

**Supplementary Figure S2 |** Resonance frequency spectra of 31 identical weak FBGs, while increasing the applied strain on the first identical weak FBG (FBG_1_).

**
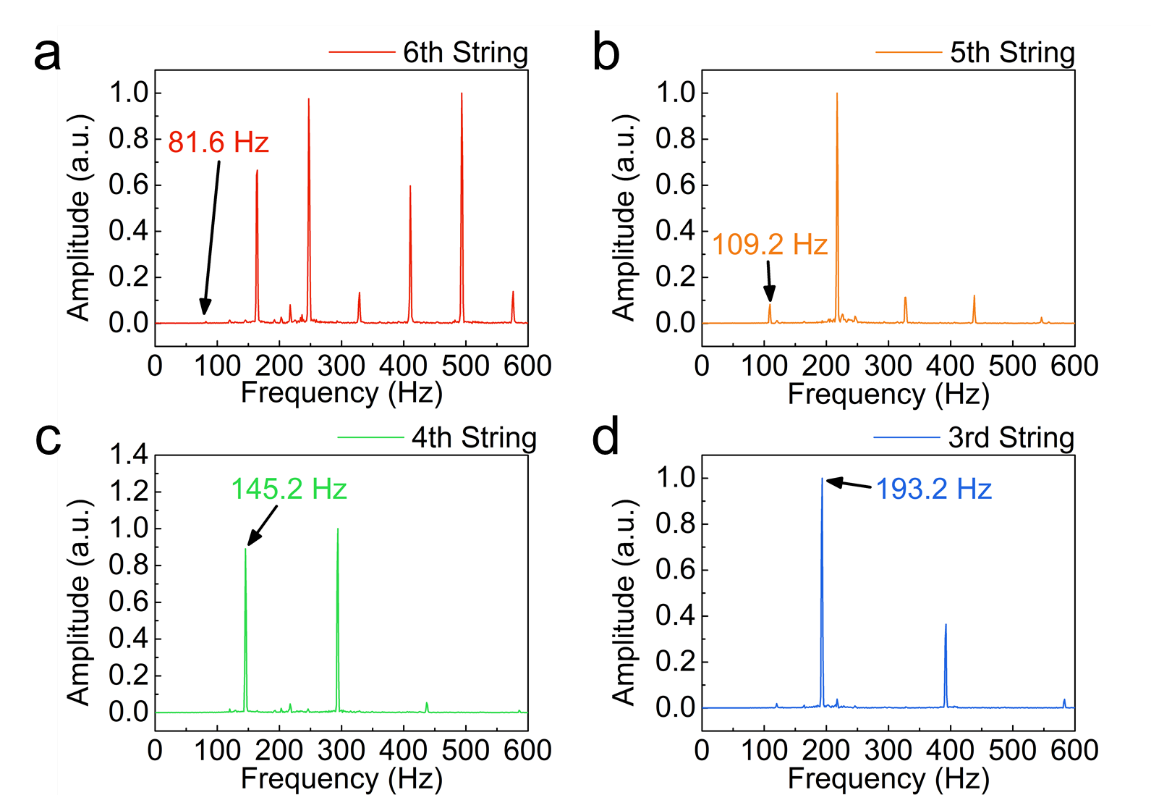
**

**Supplementary Figure S3 |** Frequency spectra of sounds of the vibrated guitar strings 6–3 (a–d, respectively) recorded by the microphone of a mobile phone (iPhone 6, Apple Inc.). Using the built-in microphone of iPhone 6 was a reliable method for the sound frequency test. However, low frequencies (below ~100 Hz) were steeply rejected owing to the optimization of speech quality. Hence, the amplitudes of the obtained first-order frequencies of the fifth and sixth strings were relatively small compared to those of the original signals^1^.

**
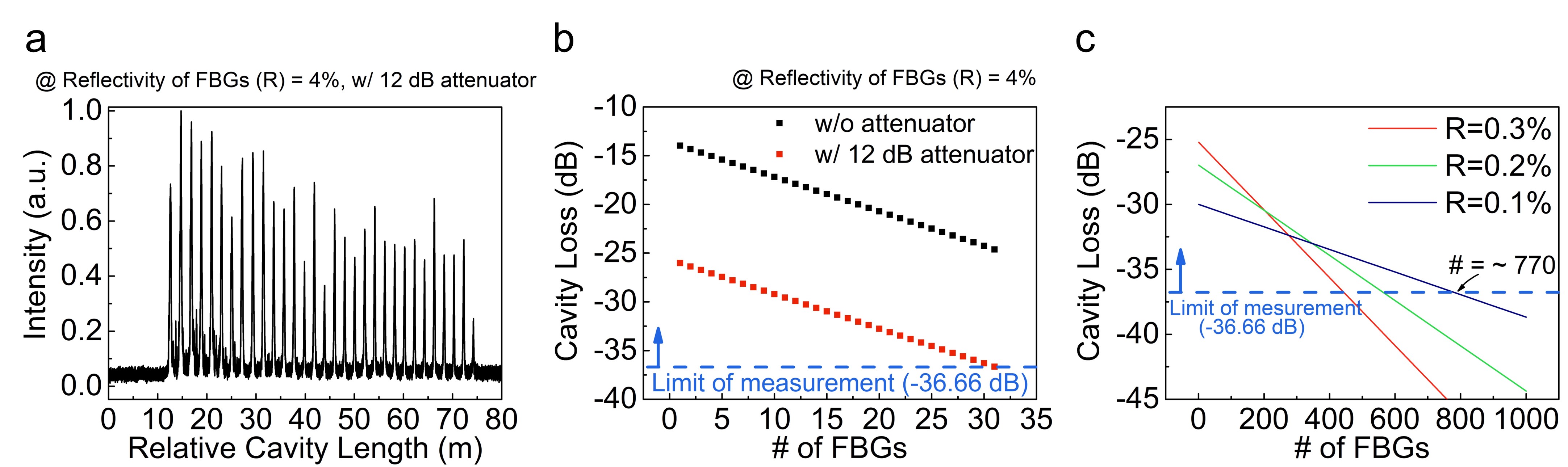
**

**Supplementary Figure S4 |** Multiplexing capacity of the proposed interrogation system based on resonance frequency mapping. (a) Resonance frequency spectrum of 31 identical weak FBGs with a reflectivity of 4%, measured by the proposed interrogation system with an added 12-dB attenuator. The 31 identical weak FBGs were successfully detected. (b) Simulation of the net cavity loss due to multiple reflections of the identical weak FBGs. By employing a 12-dB attenuator in the cavity, the net cavity loss of the 31st FBG reached −36.66 dB and was equal to the measurable limitation. (c) Simulation of cavity loss with three different reflectivities (0.3%, 0.2%, and 0.1%). Using the identical weak FBGs with a reflectivity of 0.1%, the multiplexing capacity is up to 770^2–3^.

**
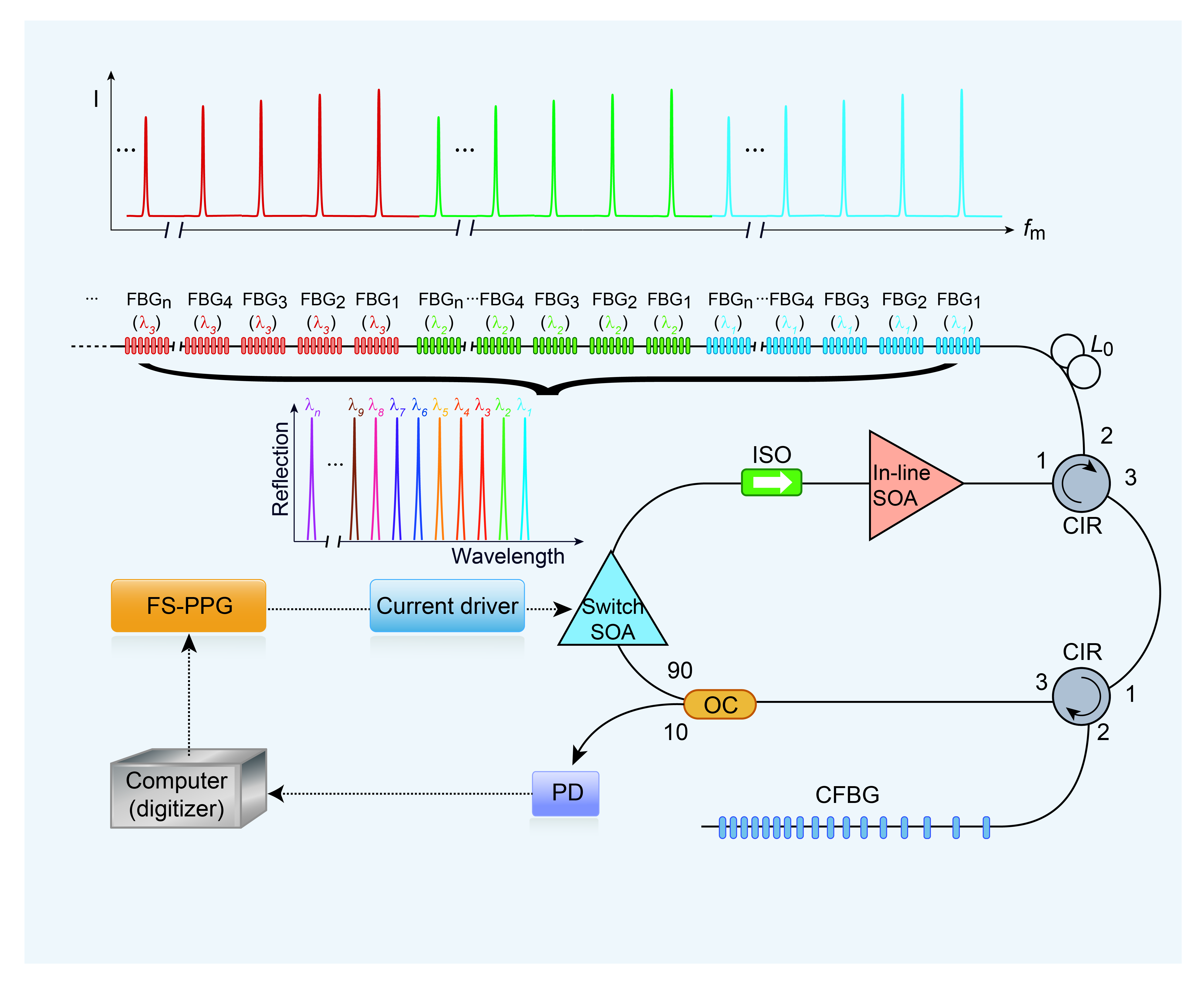
**

**Supplementary Figure S5 |** Enhanced multiplexing capacity scheme of the real-time quasi-distributed fiber optic sensor system based on resonance frequency mapping using multiple FBG arrays with slightly different center wavelengths.

**
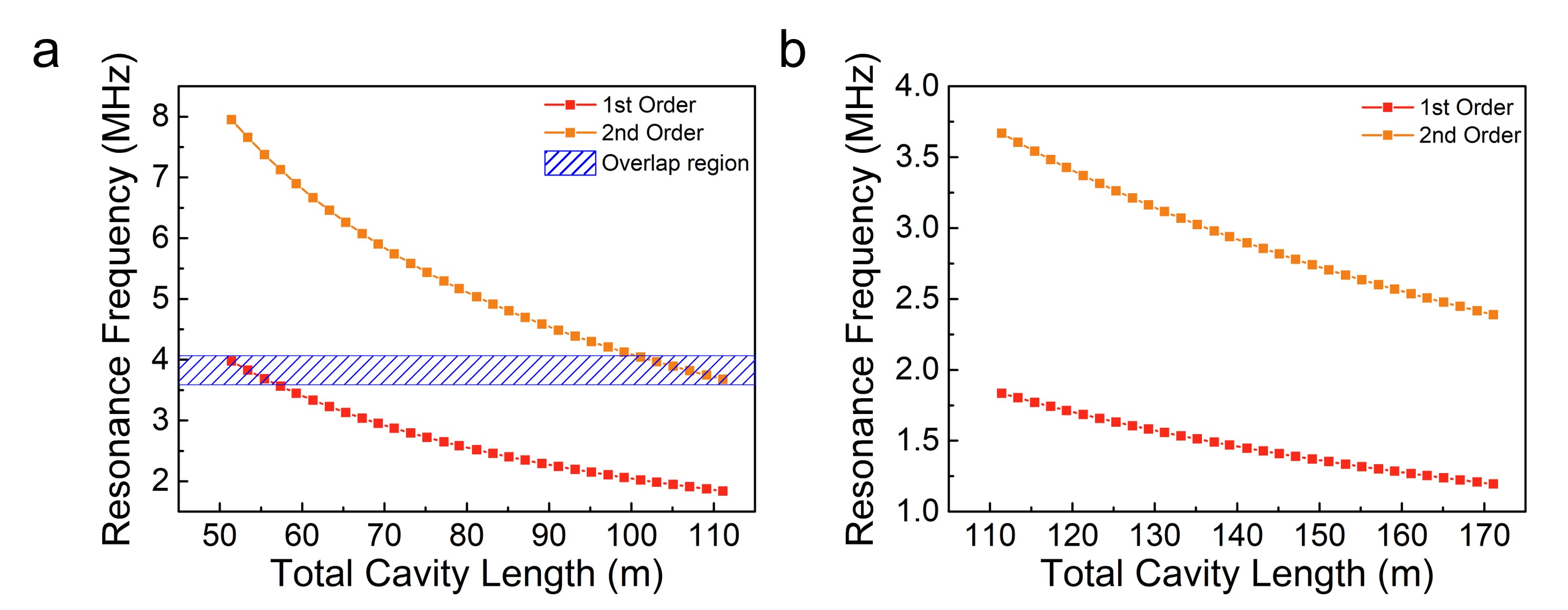
**

**Supplementary Figure S6 |** Simulation of the relationship between the resonance frequencies of 31 identical FBGs with 1-m intervals at (a) unoptimized total cavity length and (b) optimized total cavity length after adding a 30-m delayed optical fiber.


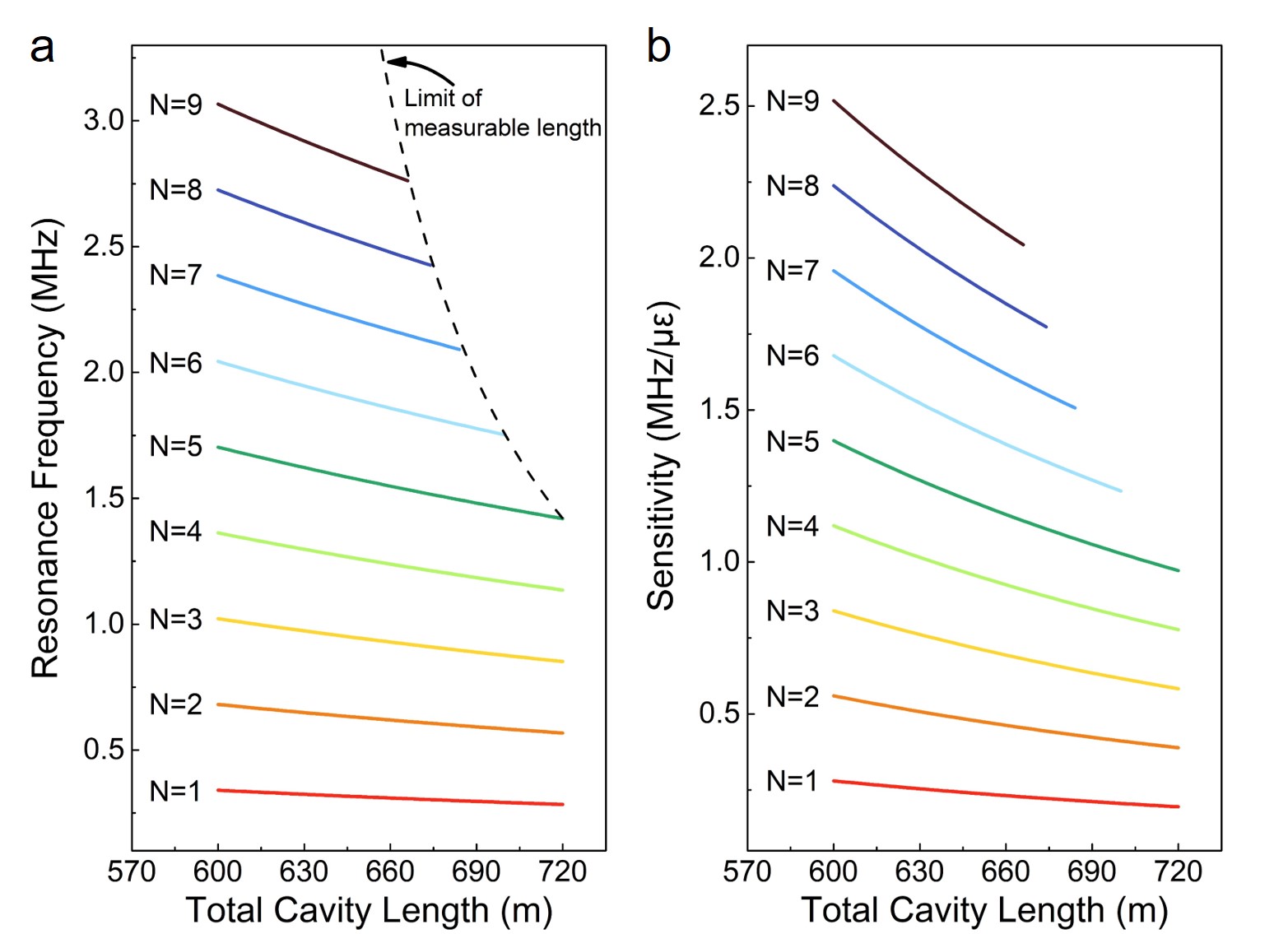


**Supplementary Figure S7 |** Simulation of (a) the measurable cavity length of the sensing head and (b) the sensitivity according to the increase in the order of resonance. The length of the sensing head was 60 m, the total cavity length of the first FBG (*L_1_*) was 600 m, and the dispersion of the CFBG was −2152 ps·nm^−1^. Under this condition, the signal overlap did not appear in the fifth order of the resonance (*N* = 5). To maximize the sensitivity, the measurement should be performed at the highest possible order of the resonance without signal overlap. Details of the measurable cavity length of the sensing head and the sensitivity can be found in Supplementary notes 1 and 2.

**
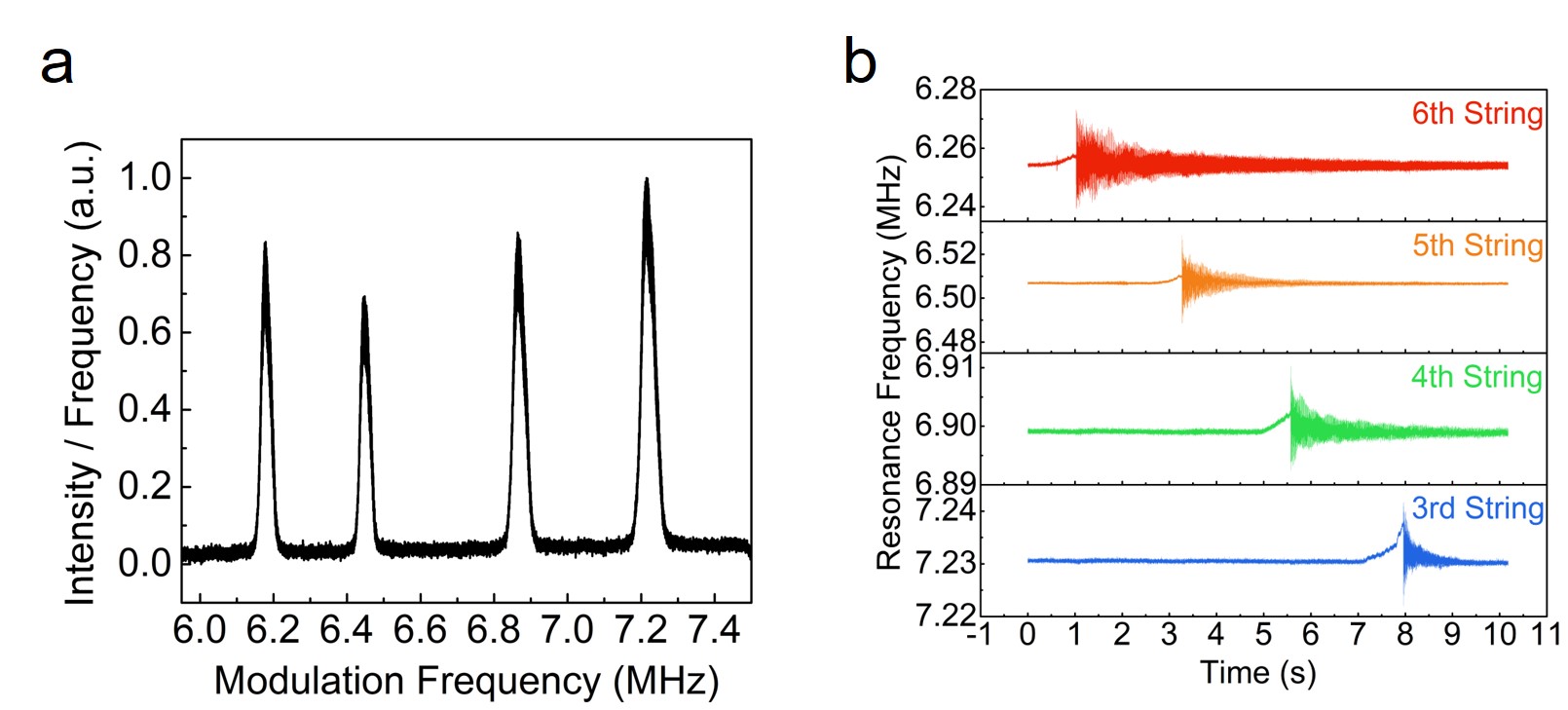
**

**Supplementary Figure S8 |** (a) Resonance frequency spectrum of four identical weak FBGs attached to the centers of the third, fourth, fifth, and sixth strings of a guitar. (b) Dynamic responses of resonance frequencies of the attached FBGs after oscillating the open guitar strings.

**Supplementary Notes**

**1. Measurable cavity length of sensing head**

According to Eqs. (2) and (3), the measurable cavity length of the sensing head can be calculated as

$L_{s}=\frac{1}{N}\cdot L_{1}$ (7)

where *L_s_* is the measurable cavity length of the sensing head, *N* is the order of the resonance, and *L_1_* is the total cavity length of the first FBG (with the shortest total cavity length among the identical weak FBG arrays).

**2. Strain sensitivity**

According to Eq. (5), the sensitivity of the change in resonance frequency to strain can be calculated as

$Sensitivity=N\cdot FSR_{i}^{2}\cdot D\cdot S$ (8)

where *N* is the order of the resonance, *D* is the chromatic dispersion of the CFBG, and *S* is the strain response constant^4^.

**Supplementary References**

1. Smith, A. Using iOS devices for noise and vibration measurements. *Sound Vib. Mag.* **51**, 14–16 (2017).
2. Wang, Y. *et al*. A quasi-distributed sensing network with time-division-multiplexed fiber Bragg gratings. *IEEE Photonics Technol. Lett.* **23**, 70–72 (2011).
3. Cooper, D. J. F., Coroy, T. & Smith, P. W. E. Time-division multiplexing of large serial fiber-optic Bragg grating sensor arrays. *Appl. Opt.* **40**, 2643 (2001).
4. Kersey, A. D., *et al.* Fiber grating sensors. *J. Light. Technol.* **15**, 1442–1463 (1997).
